# Biomarker identification of triple negative breast cancer subtypes using machine learning

**DOI:** 10.1038/s41540-026-00721-3

**Published:** 2026-04-27

**Authors:** Syed Mohammad, Vaisali Chandrasekar, Ajay Vikram Singh, Omar M. Aboumarzouk, Artefaa Al-Shamari, Sunil Choudhary, Neha Gupta, Sarada Prasad Dakua

**Affiliations:** 1 Department of Surgery, Hamad Medical Corporation, Doha, Qatar; 2 College of Health and Medical Sciences, Qatar University, Doha, Qatar; 3 Department of Chemical and Product Safety, German Federal Institute of Risk Assessment, Berlin, Germany; 4 Department of Radiotherapy and Radiation Medicine, Institute of Medical Sciences, Banaras Hindu University, Varanasi, India; 5 Department of Radiation Oncology, Apex Hospital, Varanasi, India

**Keywords:** Computational biology and bioinformatics, Biomarkers, Oncology

## Abstract

Triple Negative Breast Cancer is a clinically aggressive and molecularly heterogeneous subtype of breast cancer that currently lacks effective targeted therapies. Recognising biologically distinct subtypes within this disease is crucial for enhancing diagnosis, prognosis, and therapeutic approaches. This study introduces a comprehensive analytical framework that integrates unsupervised clustering, differential gene expression analysis, pathway enrichment, and explainable machine learning to delineate robust molecular subtypes of Triple Negative Breast Cancer and their corresponding biological mechanisms. Consensus clustering is used to divide patients into different subgroups by analyzing publicly available gene expression datasets. Pathway enrichment analysis is used to find subtype-specific gene signatures and figure out what they do. To improve interpretability and translational relevance, a model-agnostic explainable artificial intelligence approach is used to measure how much key genes and pathways help with subtype classification. The prognostic significance of the genes is further studied to demonstrate the clinical applicability of the identified biomarkers. The suggested framework works well with many different machine learning models and makes it possible to find biologically meaningful biomarkers linked to therapeutic resistance and the ability to spread cancer. These findings elucidate the molecular heterogeneity of Triple Negative Breast Cancer and endorse the advancement of more accurate and interpretable biomarker-driven clinical strategies.

## Introduction

Breast cancer is a prevalent global disease, with nearly 2.3 million new cases in 2020^1^. Triple-negative breast cancer (TNBC), an aggressive subtype lacking estrogen, progesterone, and human epidermal growth factor-2 (HER-2) expression, is associated with higher recurrence and shorter overall survival^2^. TNBC treatment becomes complicated due to the numerous subclasses and intrinsic subtypes, resulting in poor prognosis and varied clinicopathological results^3^. The primary focus of recent research has been to identify TNBC subtypes due to the inherent heterogeneity of the disease^4^. TNBC heterogeneity is inextricably linked to changes in genomic and transcriptome properties^5^. Sequencing technologies have enhanced the capacity to understand differentially expressed genes (DEG) which has facilitated the identification of clinically significant subtypes of TNBC and their relevant biomarker genes^6^. Nevertheless, despite these advancements, distant metastasis is high with a reduced survival rate, demanding early-stage biomarkers and therapeutic targets.

Numerous statistical approaches have been developed to reveal molecular subtypes using gene expression data^7,8^. Albeit such studies, there continues to be extreme diversity in TNBC tumors attributed to population-based diversity^9,10^. This mandates more reliable and universal classification approaches that could analyze the common genetic landscape driving the subclasses. The use of artificial intelligence (AI)-driven methodologies holds substantial promise in addressing these challenges, with the potential to facilitate biomarker discovery and subsequent advancements.

A significant challenge encountered in processing gene expression data stems from the intricate and high-dimensional nature of matrices representing genes versus samples. Typically, the number of samples is markedly lower than the number of genes^11^. While several deep learning (DL) and Machine Learning (ML) models have been employed for breast cancer diagnosis (Table 1), their application for TNBC is limited. This could be attributed to the high overlapping gene expression profile between the TNBC subtypes^9^. Notable studies on TNBC classification include the application of state-of-the-art (SOTA) classifiers like support vector machines (SVM), random forest (RF), decision tree (DT), and XGBoost with the boosting algorithm showing the highest performance with 99% accuracy^12,13^. However, these studies focus primarily on the binary classification between TNBC and non-TNBC and provide limited insights on the subtype analysis.

**Table 1:** Related literature on cancer classification using different feature selection approaches and several ML/DL models

Cancer types	Dataset used	Feature selection used	Classifier	Classes	Performance	Reference
Breast cancer	NCBI GEO	Pathway-based	SVM	Multiclass	94.13	Eo et al.^83^
Breast cancer	Wisconsin Breast Cancer Dataset	PCA	RF, MLP	Binary	98	Gopal et al.^84^
Breast, bladder, colon, prostate, stomach	TCGA	Mean and Standard deviation	1D-CNN	Multiclass	90	Lee et al.^24^
Breast cancer	TCGA	Pathway-based	1D-CNN, 2D-Vanilla-CNN, 2D-Hybrid-CNN	Multiclass	88.42%	Mostavi et al.^23^
Breast, colon, leukemia, lung, lymphoma,	Gems-systems.org	adaptive genetic algorithm	SVM, NB, KNN, DT	Binary	91.03	Roy et al.^80^
Prostate, lung, lymphoma	Ico2s.org, NCBI GEO	Hybrid L1/2+2 regularisation	SVM	Binary	99.87	Huang et al.^85^
Breast	NCBI GEO	ReliefF	Native Bayers	Binary	89	Bolon-Canedo et al.^45^
Breast	NCBI GEO	Gain ratio	KNN	Binary	91.96	Akpinar and Oduncuoglu^86^
Breast	NCBI GEO	–	SVM-RFE	Binary	86.09	Li et al.^87^
Acute myeloid leukemia	Microarray		ANN, SVM, NB, regression, KNN, DT	Binary	98	Dwivedi et al.^21^
TNBC	NCBI GEO	DEG	SVM, KNN, DT, XGBoost	Binary	99	Thalor et al.^13^
TNBC	NCBI GEO	DEG	SVM	Multiclass	98.8	Bissanum et al.^15^
TNBC	TCGA and GEO	DEG	RF	Binary	76	Chen et al.^88^
TNBC	GEO	Fisher score and Chi-2	RF, Gradient boost, XGboost	Multiclass	99	Azzouz et al.^89^
TNBC	TCGA	DEG	RF	Binary	100	Chen et al.^90^
TNBC	TCGA	Gene correlation neural network	SVM, RF, XgBoost, KNN, DT, LR	Multiclass	98.9	Liu et al.^27^

(Only performances of the SOTA predictors are provided).

Generally, statistical analysis of DEG is used for feature selection^14^. However, the inherent heterogeneity among TNBC subtypes necessitates the integration of DEG analysis with other feature selection techniques^15^. Among the different methods, the filter approach has been proven to be effective for high-dimensional data^16^. For instance, the application of the Pearson correlation coefficient (PCC) to select optimized features resulted in an accuracy of 93.1% using a deep convolutional neural network (CNN)^17^. Similarly, a two-stage gene selection process predicted the class with 89% accuracy using a SVM classifier^18^. While other feature selection techniques are equally effective (Table 1), it is important to incorporate prior knowledge of gene expression into feature selection^19^. Like feature selection algorithms, the choice of classifiers also plays an equivalent role in prediction accuracy. Hence, leveraging SOTA ML models enables harnessing the power of these refined feature sets to predict subtypes and identify the genetic signature.

Numerous researchers have previously introduced diverse techniques over the years, employing a spectrum of ML algorithms across various breast cancer data repositories. The performance of each model exhibits variations dependent on the algorithm employed and the specific dataset. Supervised and unsupervised are presently the two most commonly utilized strategies in cancer categorization. Recent studies have used feature selection approaches and ML to find sex-specific colorectal cancer biomarkers^20^. While some studies have progressed to employing complex DL algorithms^21^, ML models continue to hold more promise in analyzing gene expression data owing to their interpretable nature, better data handling possibilities, opportunities for feature ranking, and high robustness in the case of noisy gene expression data^22^.

When dealing with gene expression data, it becomes crucial to account for the specific challenges posed by the data high dimensionality. This poses a significant challenge due to the limited number of samples, creating a risk of overfitting. While DL methods like CNN show classification accuracy of 88.4% in breast cancer classification using TCGA data^23,24^, conventional ML models like SVM and Naïve Bayes (NB) with tailored feature selection techniques show an accuracy of 91%^18^. Specific to TNBC classification, limited reports exist in the application of ML models, as most studies continue to look into traditional statistical approaches to cluster and classify TNBC^25,26^. Among the few TNBC classifications using ML, RF and SVM have been the most popular models (Table 1). Several studies report high accuracy in TNBC classification with 99–100% accuracy (Table 1). Similarly, Liu et al.^27^ employ a DL-based feature selection approach and test its effectiveness using SVM, RF, XGBoost, KNN, and DT, resulting in an accuracy of 84.9%, 91%, 89.9%, 64.9%, and 74.8%, respectively^27^. However, these studies fail to consider several factors like data consistency across demographics, data imbalance or multiclass identification. Such SOTA models, often striking a balance between complexity and performance, are well-suited for datasets with small sample sizes like in the most commonly used NCBI and TCGA datasets. Furthermore, opting for SOTA models without unnecessary complexities ensures a streamlined approach that enhances model interpretability. Based on such extensive literature, classifiers like SVM, DT, XGBoost, and RF have been found to be best suited for gene expression analysis.

Albeit the availability of several classifiers, the application of ML models for TNBC classification is limited. This could be attributed to the inconsistency that arises from data non-uniformity and complexity, which necessitates domain-specific expertise. Previous research has discovered several genes associated with TNBC, with some having distinct expression patterns in specific nations, while some lack uniformity^13,28^. However, to our knowledge, no such study exists in which common genes from diverse populations have been identified and evaluated for their role in TNBC progression. As a result, this study aims to elucidate the shared genetic signature among TNBC from diverse demographics. The goal is to build resilient models capable of recognizing the genetic markers of TNBC subtypes, with a focus on identifying common genes that show differential expression in cancer samples regardless of demographic differences. To achieve this goal, we employ clustering algorithms to categorize the amalgamated TNBC expression data. Another contribution of this study is the application of a specific feature selection approach incorporating domain-specific inputs and gene correlation analysis. This approach aims to derive universal TNBC biomarkers through gene ontology studies and pathway enrichment analysis. The current study aims to leverage the simplicity, interpretability, and robustness of SOTA ML models with appropriate feature selection approaches for TNBC subtype prediction. To the best of our knowledge, this study is among a few on the interpretability aspect of the AI model with TNBC classification based on subtypes derived from a clustering algorithm. Lastly, the validation of the feature selection approach and interpretability is performed using pathway enrichment analysis. This step is crucial for decoding the phenotypic manifestations of TNBC subtypes, contributing to a deeper understanding of pathogenesis and facilitating biomarker identification. In summary, the current work not only addresses existing gaps in TNBC classification but also introduces novel methodologies to enhance the interpretability and applicability of ML models in this context.

The remainder of the work is organised as follows: The “Results” section describes the results achieved and the “Discussion” section talks about the interpretation of the results. The “Methodology” section describes the proposed workflow and methodology adapted at each stage of the work.

## Results

Multiple NCBI GEO datasets are amalgamated to encompass a diverse population and substantial TNBC samples. Six NCBI GEO datasets are combined after careful screening of the database to obtain a robust sample size of TNBC samples. Six GEO datasets are normalised to obtain a final dataset of 807 samples with 565 TNBC and 242 non-TNBC samples. The microarray gene expression profile for these samples encompasses 23,520 genes, which are the features representing different sub-classes.

In the next step, the gene expression data are clustered to identify the molecular subtypes among the TNBC samples to better understand the dataset and the subtypes as well. Two clustering approaches are adapted, namely K-means^29,30^ and hierarchical clustering.

**Fig. 1:**
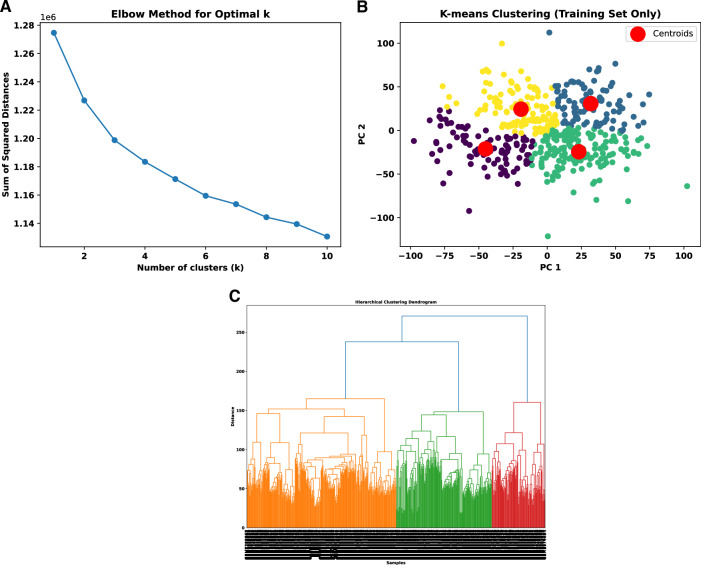
Unsupervised clustering approaches used to identify molecular subtypes of TNBC. **A** Elbow method plot showing the sum of squared distances across a range of *k*-values to determine the optimal cluster count. **B** Principal Component Analysis (PCA) scatter plot of the K-means clustering results, where red circles denote the centroids for each identified group. **C** Hierarchical clustering dendrogram illustrating the structural relationship and distance between samples, with branches color-coded to represent distinct molecular groupings.

K-means and hierarchical clustering are performed on the combined and pre-processed Geo gene expression dataset to identify the gene expression signature. The elbow method is employed to determine the value of K (Fig. 1A); the acceptable number of clusters is found to be four (Fig. 1B) and is quite stable. As the aim of the current study is to identify subtypes and their genetic signatures, K-means clustering is found to be effective, resulting in four stable clusters similar to Burstein subtypes^28^ and the redefined Lehman type^31^. Within the four identified TNBC subtypes, 72 samples belonged to subtype C1, 101 to C2, 129 to C3, and 150 to C4. In addition, the non-TNBC group (designated as subtype C0) consisted of 242 samples.

Similar to K-means clustering, the bottom-up agglomerative clustering approach indicates similar performance, resulting in a similar number of clusters (Fig. 1C). Visualisation of the clusters and their separation is obtained using principal component analysis (PCA) (Fig. 1B). Both clustering algorithms identify four distinct subtypes similar to the Burstein subtype^28^. We further attempt to compare the clusters with the Burstein subtypes. Cluster C3 indicates higher similarity towards the LAR subtype, whereas C1 and C4 show similarity between both BLIS and BLIA of the Burnstein types, respectively.

### Gene/feature selection

**Fig. 2:**
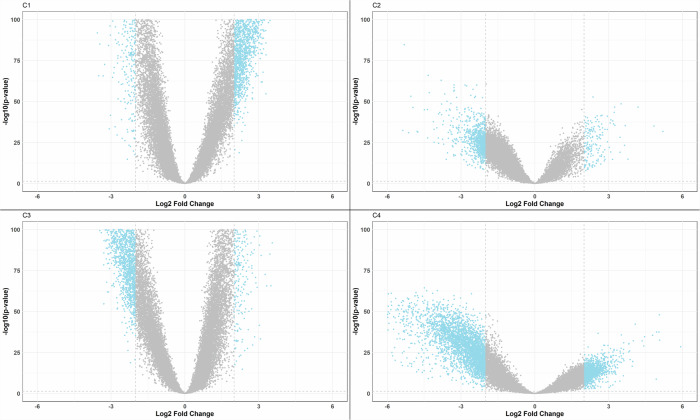
Volcano plots illustrating differentially expressed genes across TNBC subtypes. Panels **C1** through **C4** display the distribution of gene expression changes for each respective subtype. The *x*-axis represents the *Log*_2_ Fold Change, and the *y*-axis represents the statistical significance (−*log*_10_ *p*-value). Blue data points indicate significantly upregulated or downregulated genes that meet the study’s threshold for biomarker selection.

The differential expression of genes is analyzed for each cluster using fold change > 2 with significance at adjusted (FDR or Benjamini–Hochberg correction) *p* < 0.05. The results of the differential gene expression for a single subtype/cluster against other subtypes as well as non-TNBC samples are presented in the form of volcano plots (Fig. 2). The four clusters indicate 313, 248, 139, and 244 uniquely DEG in each cluster. The unique DEGs across each of the subtypes are combined, resulting in 944 genes across all clusters that are used for model development and testing.

To identify the most influential genes for pathway analysis, we employ RF feature importance, which effectively ranks genes based on their contribution to the predictive model. This method captures complex gene interactions without assuming linearity, ensuring robustness in high-dimensional and noisy gene expression data. To select the most informative genes for model development, we have employed RFE that optimizes the subset for ML by retaining only the most informative predictors.

### Gene ontology, reactome analysis, canonical pathway analysis

**Fig. 3:**
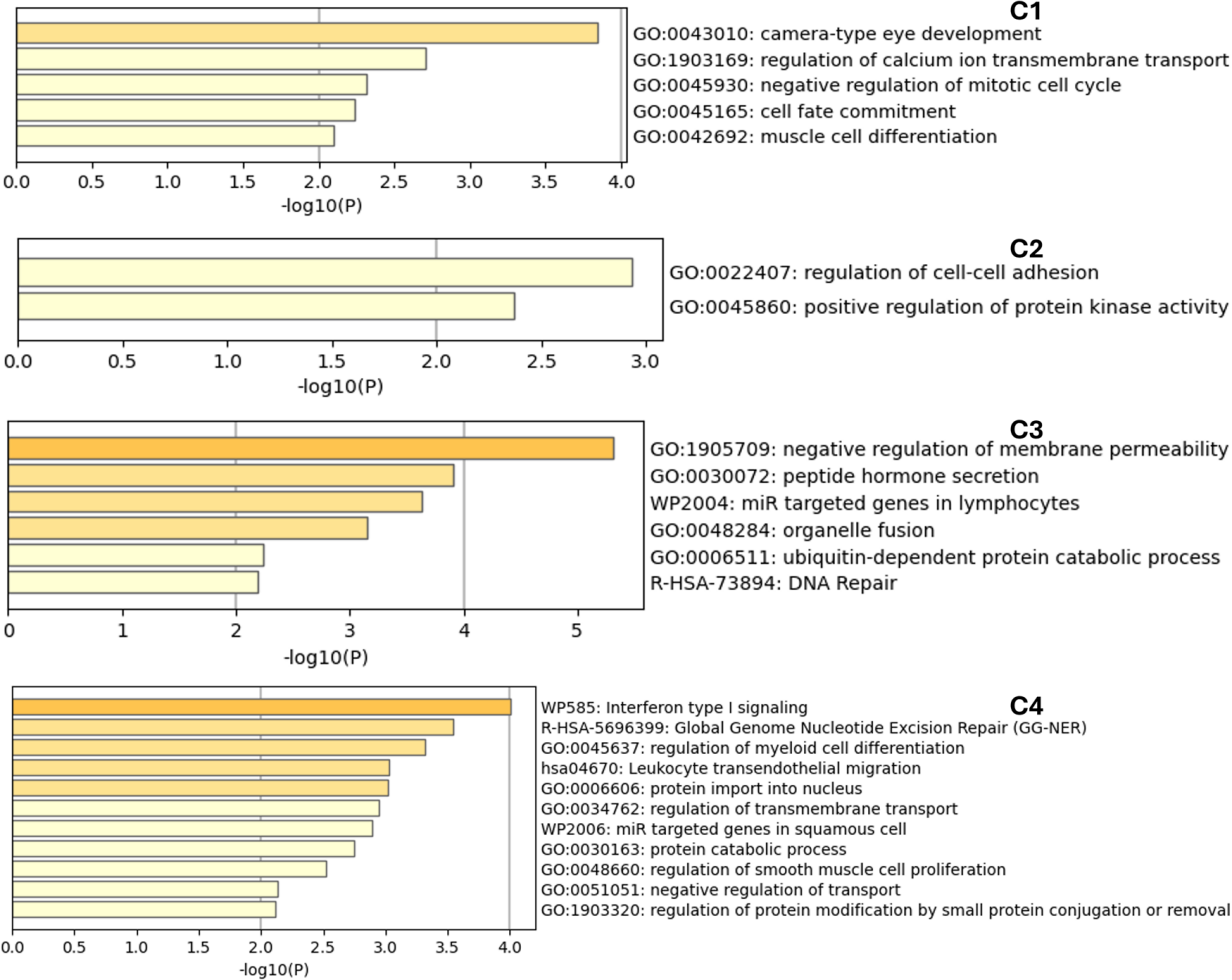
Pathway enrichment analysis of TNBC molecular subtypes. This figure illustrates statistically significant GO and Reactome pathways enriched across the four TNBC clusters (**C1**–**C4**). Pathways were identified based on differentially expressed genes using over-representation analysis, with significance determined by adjusted *p*-values. Distinct biological processes related to development, metastasis, immune signaling, and therapy resistance are highlighted across subtypes.

The results of the statistically enriched genes (GO/Reactome) are presented in Fig. 3. It may be noted that these enriched pathways are identified with significant *p*-values.

The results of enrichment analysis for the C1 cluster indicate that unique DEGs in camera-type eye development, regulation of calcium ion transmembrane transport, negative regulation of mitotic cell cycle^32^, muscle cell differentiation, etc. (Fig. 3).

In the case of subtype C2, only two pathways were found to be enriched, viz., regulation of cell-cell adhesion and positive regulation of protein kinase activity 3. Considering the limited pathways for the C1 and C2 subtypes, further Reactome analysis is performed (Tables 2 and 3). The lateral plate mesoderm and paraxial mesoderm pathway involvement in C1 subtype indicates the involvement of the epithelial-to-mesenchymal transition (EMT)^33^, like cancer progression.

**Table 2:** Top most relevant pathways with *p*-value < 0.05 in C1 subtype (FDR-False Discovery Rate)

Pathway name	Entities			Reactions	
	Found	*p*-value	FDR	Found	Ratio
Formation of lateral plate mesoderm	2/22	0.001	0.596	3/5	3.28e-04
Formation of paraxial mesoderm	3/260	0.011	0.596	5/29	0.002
LGI-ADAM interactions	1/14	0.031	0.596	1/5	3.28e-04
Calcitonin-like lignad receptors	1/17	0.038	0.596	3/8	5.24e-04
Packaging of eight RNA segments	1/26	0.058	0.596	1/2	1.31e-04
Hormone ligand-binding receptors	1/26	0.058	0.596	2/8	5.24e-04

**Table 3:** Top most relevant pathways with *p*-value < 0.05 in C2 subtype (FDR-False Discovery Rate)

Pathway Name	Entities			Reactions	
	Found	*p*-value	FDR	Found	Ratio
NR1H2 and NR1H3 regulate gene expression linked to triglyceride lipolysis in adipose	2/11	3.97e-04	0.119	2/2	1.31e-04
Defective SLCO2A1 causes primary, autosomal recessive hypertropic osteoarthropathy 2 (PHOAR2)	1/5	0.013	0.633	1/1	6.55e-05
Progressive trimming of alpha-1,2-linked mannose residues from Man9/8/7GlcNAc2 to produce Man5GlcNAc2	1/10	0.026	0.633	4/11	1.97e-04
Platelet degranulation	3/250	0.028	0.633	4/11	7.21e-04
Terminal pathway of complement	1/13	0.033	0.633	5/5	3.28e-04
Arachidonate production from DAG	1/16	0.041	0.633	1/3	1.97e-04
Ion influx/efflux at host-pathogen interface	1/19	0.048	0.633	1/3	1.31e-04
Regulation of gap junction activity	1/20	0.051	0.633	1/2	2.62e-04
Response to elevated platelet cytosolic Ca2+	1/332	0.056	0.633	4/14	9.17e-04

Analysis of enriched GO and KEGG pathways for C3 and C4 shows the involvement of cancer metastasis and therapy resistance, but through the involvement of different pathways. For instance, enrichment of membrane permeability regulation and peptide hormone secretion (Fig. 3) indicates the possibility of metastasis through vascular permeability, circulating tumor cells that could invade stromal cells due to enhanced vascular permeability^34^.

Similarly, interferon type 1 signaling is enriched in the C4 cluster; several studies have explored the involvement of the STimulator of INterferon Genes (STING) pathway in TNBC^35,36^. A closer examination of the Reactome pathways that are affected in the C4 cluster indicates the involvement of autophagy (a cellular response to stimuli), RNA metabolism, transcription, transport mechanisms, and signal transduction.

### TNBC classification by ML models

**Fig. 4:**
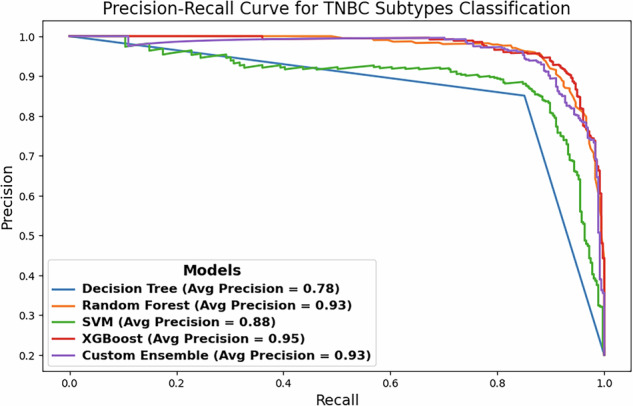
Precision-recall curves illustrating the performance of four machine learning models. Precision-Recall (PR) curves for five different machine learning architectures: Decision Tree (blue), Random Forest (orange), SVM (green), XGBoost (red), and a Custom Ensemble (purple). The XGBoost model achieved the highest Average Precision (AP) of 0.95. The curves plot Precision against Recall across various thresholds, highlighting the trade-offs in classification performance for the TNBC subtypes.

**Fig. 5:**
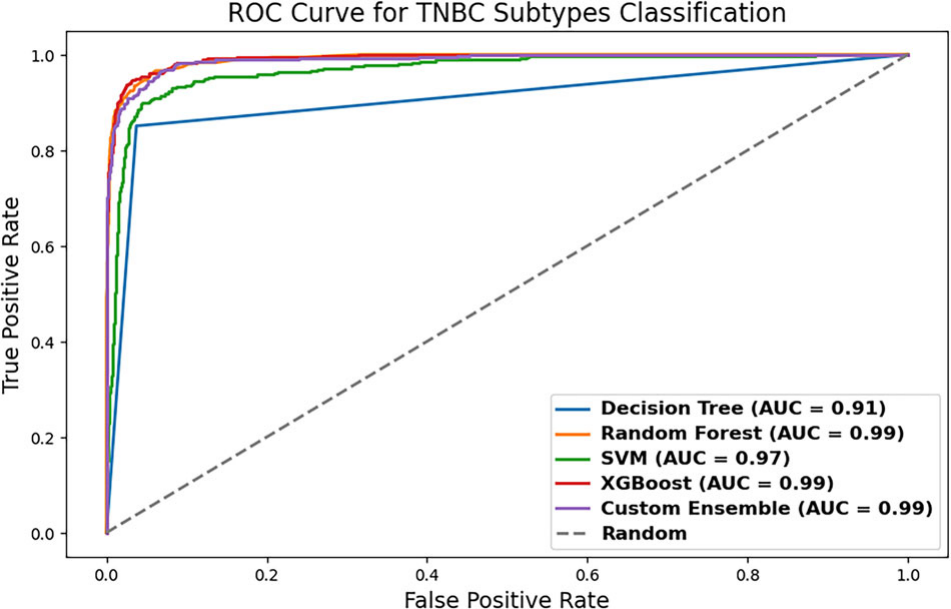
Receiver operating characteristic curves illustrating the predictive performance of four machine learning models. ROC curves comparing the performance of the Decision Tree (AUC = 0.91), SVM (AUC = 0.97), and the Random Forest, XGBoost, and Custom Ensemble models (all AUC = 0.99). The plot displays the True Positive Rate (Sensitivity) against the False Positive Rate (1-Specificity). The dashed gray line represents the performance of a random classifier, serving as the baseline for model evaluation.

Initially, the models are trained with the features obtained after DEG analysis to get the baseline performance of the models. Four different models of three different categories, viz., vector-based, tree-based, and boosting algorithms, are trained for this multiclass classification. The baseline performance of the models chosen for the classification task is shown in Table 4. In the current study, we analyze prediction performance using a precision-recall curve (Fig. 4) along with TPR vs FPR Receiver Operating Characteristic curve (Fig. 5).

**Table 4:** Performance summary of ML models before and after feature correlation and selection strategy

Baseline							After feature correlation and selection						
Model	Class	Accuracy	Sensitivity	Specificity	Precision	F1	Model	Class	Accuracy	Sensitivity	Specificity	Precision	F1
Random forest	0	0.96	0.97	0.92	0.96	0.96	Random forest	0	0.98	0.98	0.97	0.99	0.98
	1	0.92	0.81	0.95	0.81	0.81		1	0.98	0.88	0.99	0.74	0.80
	2	0.94	0.50	0.98	0.78	0.61		2	0.95	0.43	0.99	0.86	0.57
	3	0.94	0.71	0.93	0.71	0.71		3	0.96	0.81	0.98	0.83	0.82
	4	0.94	0.86	0.96	0.73	0.79		4	0.94	0.89	0.95	0.67	0.76
Decision tree	0	0.92	0.95	0.87	0.94	0.94	Decision tree	0	0.92	0.96	0.87	0.94	0.95
	1	0.93	0.75	0.98	0.67	0.70		1	0.98	0.75	0.99	0.75	0.75
	2	0.92	0.50	0.96	0.54	0.52		2	0.93	0.50	0.97	0.56	0.53
	3	0.94	0.52	0.98	0.80	0.63		3	0.95	0.61	0.98	0.79	0.69
	4	0.92	0.74	0.94	0.62	0.67		4	0.91	0.66	0.94	0.57	0.61
Support vector machine	0	0.92	0.96	0.81	0.91	0.93	Support vector machine	0	0.92	0.96	0.83	0.92	0.94
	1	0.97	0.75	0.98	0.71	0.73		1	0.97	0.69	0.99	0.79	0.74
	2	0.94	0.54	0.97	0.68	0.60		2	0.95	0.57	0.98	0.76	0.65
	3	0.95	0.65	0.98	0.77	0.70		3	0.93	0.55	0.98	0.68	0.62
	4	0.93	0.63	0.96	0.68	0.65		4	0.93	0.74	0.95	0.65	0.69
Gradient boosting	0	0.95	0.92	0.97	0.93	0.92	Gradient boosting	0	0.98	0.97	0.98	0.99	0.98
	1	0.91	0.77	0.94	0.93	0.84		1	0.98	0.94	0.99	0.75	0.83
	2	0.91	0.59	0.99	0.93	0.64		2	0.95	0.57	0.99	0.80	0.67
	3	0.95	0.93	0.92	0.93	0.93		3	0.97	0.81	0.99	0.86	0.83
	4	0.91	0.83	0.96	0.93	0.87		4	0.95	0.87	0.96	0.70	0.78
Custom built ensemble	0	0.92	0.90	0.97	0.93	0.91	Custom built ensemble	0	0.97	0.98	0.97	0.99	0.98
	1	0.90	0.81	0.94	0.85	0.83		1	0.99	0.88	0.99	0.82	0.85
	2	0.92	0.56	0.99	0.75	0.64		2	0.95	0.54	0.98	0.75	0.63
	3	0.90	0.85	0.93	0.85	0.85		3	0.97	0.81	0.98	0.83	0.82
	4	0.91	0.83	0.96	0.86	0.85		4	0.94	0.87	0.95	0.67	0.76

The initial baseline models exhibit high values against each metric (Table 4). All the models perform relatively well for all classes, which is well in accordance to several published reports on cancer recognition accuracy using ML models^37,38^. Tree-based models like DT and RF show low sensitivity for one of the minority class, C2. In contrast, SVM and boosting-based algorithms achieved comparatively higher sensitivity values. However, even for these models, the sensitivity plateaus at approximately 0.59 (Table 4). A similar trend is observed for TNBC classification into Burnstein subtypes, when several ML models are used for classification^15^. Alternatively, the other models show extremely high specificity for the class with fewest data points, indicating model overfitting. Interestingly, this custom ensemble indicates high performance in predicting all subtypes.

**Fig. 6:**
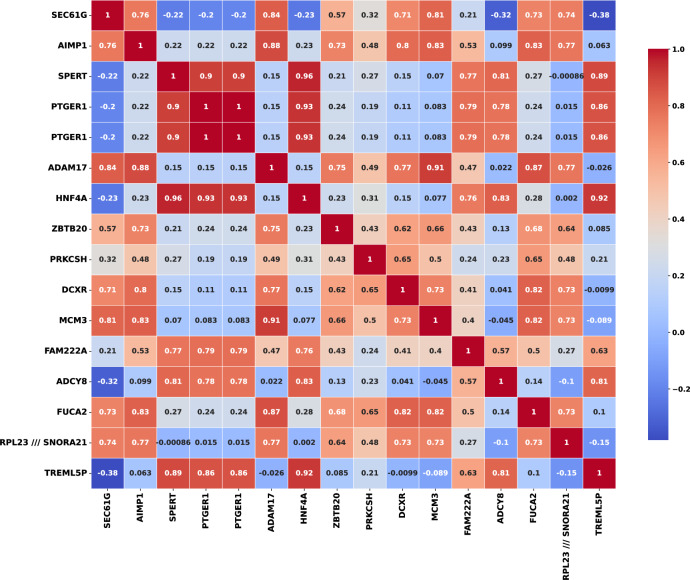
Feature correlation heat map illustrating pairwise relationships among selected gene features. Heatmap showing the Pearson correlation coefficients between the expression levels of 30 representative genes. The color scale on the right indicates the strength of the relationship, ranging from blue (negative correlation) to red (positive correlation). Each cell contains the specific correlation value calculated for the corresponding gene pair on the *x* and *y* axes.

In this study, we utilize a two-step approach: feature correlation analysis (FCA) to remove highly correlated (Fig. 6) and redundant features^39^, followed by recursive feature elimination (RFE)^40^ with a RF-based model to identify the most informative genes for TNBC classification.

**Fig. 7:**
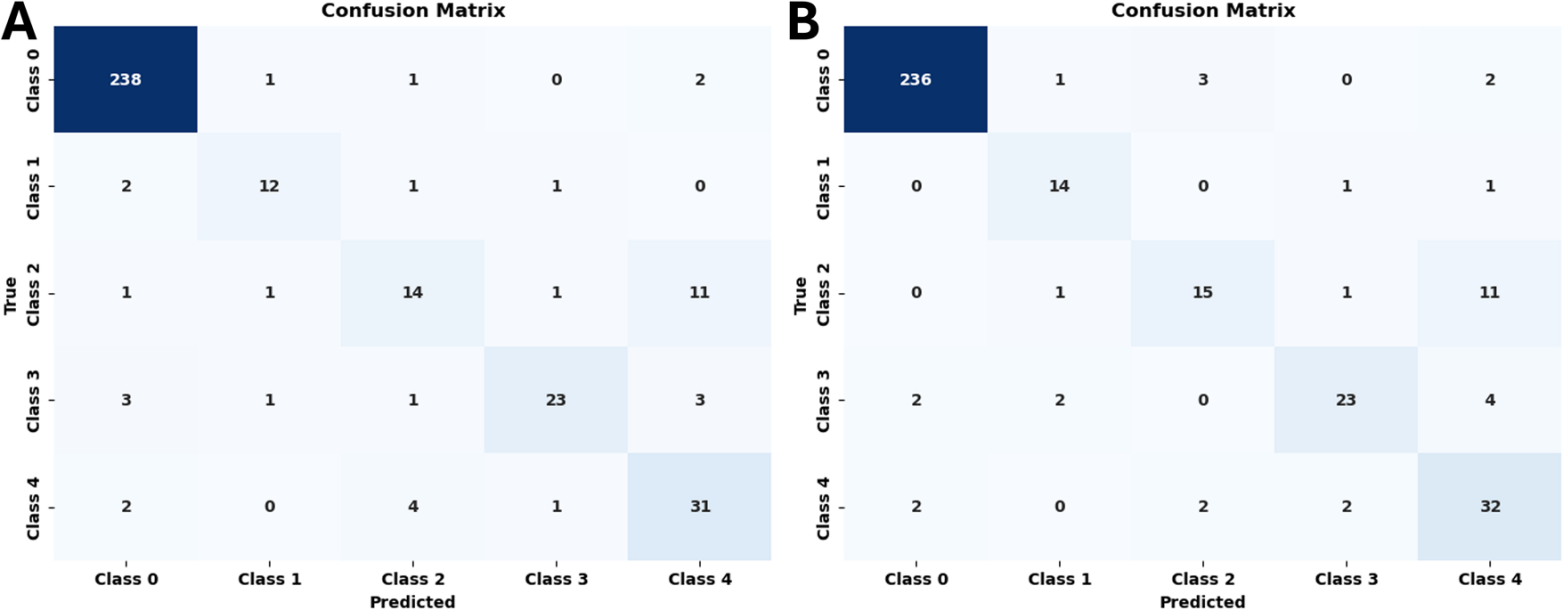
Confusion matrices of the custom voting ensemble model before and after feature refinement. Confusion matrix for the Custom voting ensemble model **A** before feature correlation and Recursive feature elimination and **B** after feature correlation and Recursive feature elimination. In both panels, rows represent the ground-truth “True” classes, and columns represent the “Predicted" classes for Subtypes C0 through C4. The numerical values and color intensity in the diagonal cells indicate the number of correctly classified samples, while off-diagonal cells show the distribution of classification errors.

The impact of these techniques is evident in the subsequent model evaluations (Table 4 and Fig. 7). Post-feature refinement/removal of highly correlated features and feature selection, the models demonstrate improved generalization, as evidenced by reduced overfitting. The impact of RFE is also evident in the post-feature-selection results, where accuracy and F1 have improved for harder-to-classify classes, such as Class 2; the accuracy has increased from a maximum of 0.94 to 0.96, and the F1-score has improved from 0.64 to 0.67. Accuracy has remained stable, with slight improvements in certain classes, such as Class 1 (from 0.92 to 0.98). Precision has also improved for some classes (e.g., Class 1 increased from 0.71 to 0.79), highlighting the model’s enhanced ability to correctly identify subtype-specific gene expression patterns.

Additionally, the overall model’s performance is assessed using Receiver Operating Characteristic - Area Under Curve (ROC-AUC) (Fig. 5) and Precision-Recall (Fig. 4) curves. The AUC values highlight the strengths of different classifiers, with RF and XGBoost (AUC = 0.99) along with the custom ensemble (AUC = 0.99) ranking the highest. The DT model, despite its simplicity, demonstrates impressive performance (AUC = 0.91). This is similar to several studies published on gene expression-based prediction^41^. While this simplicity is advantageous for understanding the decision-making process, it fails to capture the complex relationships within the gene expression data. Similarly, in the Precision-Recall analysis, XGBoost has achieved the highest average precision (0.95), followed closely by RF and the Custom Ensemble model (both 0.93), indicating their superior ability to correctly classify the TNBC subtypes while maintaining high recall.

The custom-built ensemble classifier, utilizing a hard voting system to combine predictions from DT, RF, SVM, and XGBoost, is also one of the top-performing models, unlike individual models. With an accuracy and AUC-ROC score as high as 0.99, sensitivity as high as 0.98, along specificity and precision reaching 0.99, the ensemble ones effectively harness the strengths of their constituent classifiers. Many studies have reported ensemble machine learning models to show superior performance, especially with complex data such as gene expression, as they are able to effectively combine the strengths of all its constituent classifiers^42,43^.

**Fig. 8:**
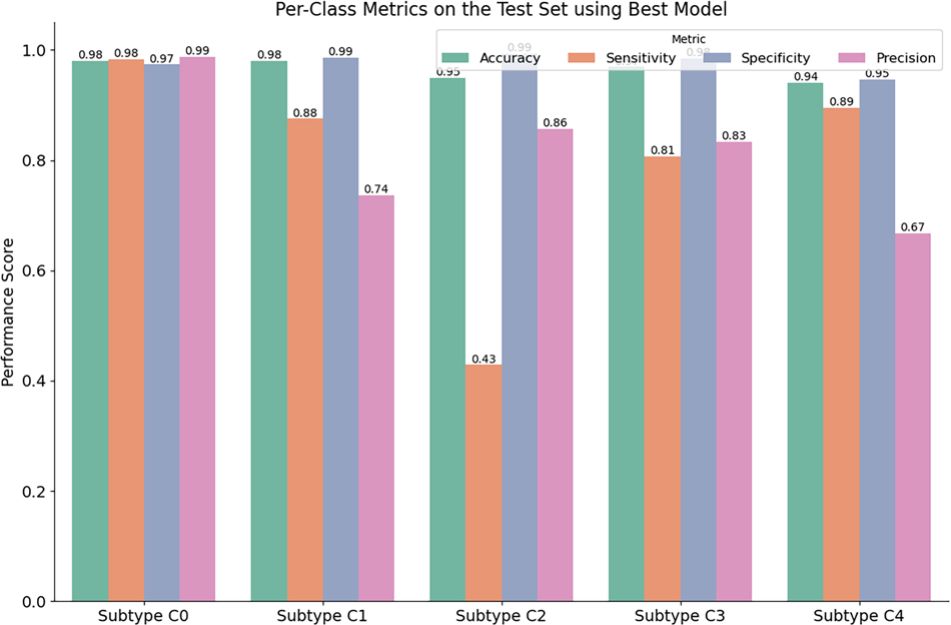
Performance comparison of the TNBC subtypes across several metrics. Bar plot displaying per-class metrics on the test set for subtypes C0, C1, C2, C3, and C4. The *y*-axis represents the performance score ranging from 0.0 to 1.0. Four metrics are evaluated for each subtype: Accuracy (teal), Sensitivity (orange), Specificity (blue), and Precision (pink). Specific numerical values are annotated above each bar to provide precise performance measurements.

Compared to the overall model performance, the classification results highlighted in Fig. 8 reveal notable differences among TNBC subtypes. While accuracy remains consistently high (0.90–0.99), the sensitivity varies significantly, with C0 and C3 achieving strong recall (0.81–0.98), whereas C2 shows much lower sensitivity (0.43). Specificity, on the other hand, remains high across all the subtypes, particularly for C2 (0.99), indicating that the model reliably excludes misclassified cases for this subtype. Precision follows a similar trend, with C0 achieving one of the highest precision (0.99), C2 also having high precision (0.86), while C1, C3, and C4 exhibit higher false positive rates.

**Fig. 9:**
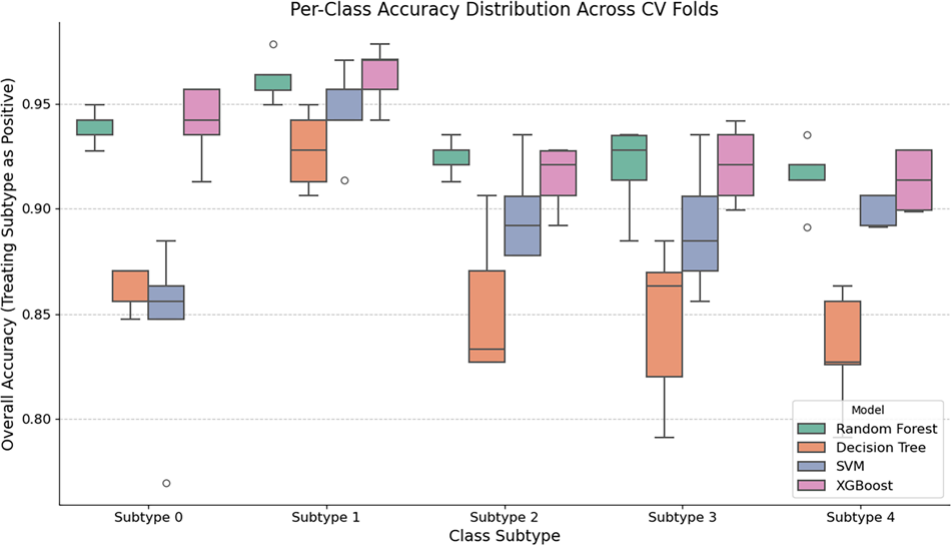
Per-class accuracy distributions of machine learning models across TNBC subtypes. Box plot comparing the accuracy of different machine learning models (RF, DT, SVM, and XGBoost) across TNBC subtypes over 10 independent runs of stratified train-test splits. The models are evaluated on multiple splits to assess the stability and generalizability of their performance.

Building on the subtype-wise performance comparison, we further evaluate model-specific variations by training multiple ML classifiers such as RF, DT, SVM, and XGBoost over repeated runs (Fig. 9). This approach allows to assess not only the overall accuracy of each model but also the model consistency across TNBC subtypes. By standardizing the dataset and applying stratified train-test splits, we have ensured balanced subtype representation, minimizing biases in the evaluation.

The results indicate that the ensemble models, particularly RF and XGBoost, demonstrate superior and more stable accuracy^42,43^ across subtypes, particularly in Subtypes 0, 2, and 4. SVM also performs consistently well, whereas DT shows the highest variability and lower accuracy in certain subtypes, highlighting its tendency to overfit.

### SHAP analysis

**Fig. 10:**
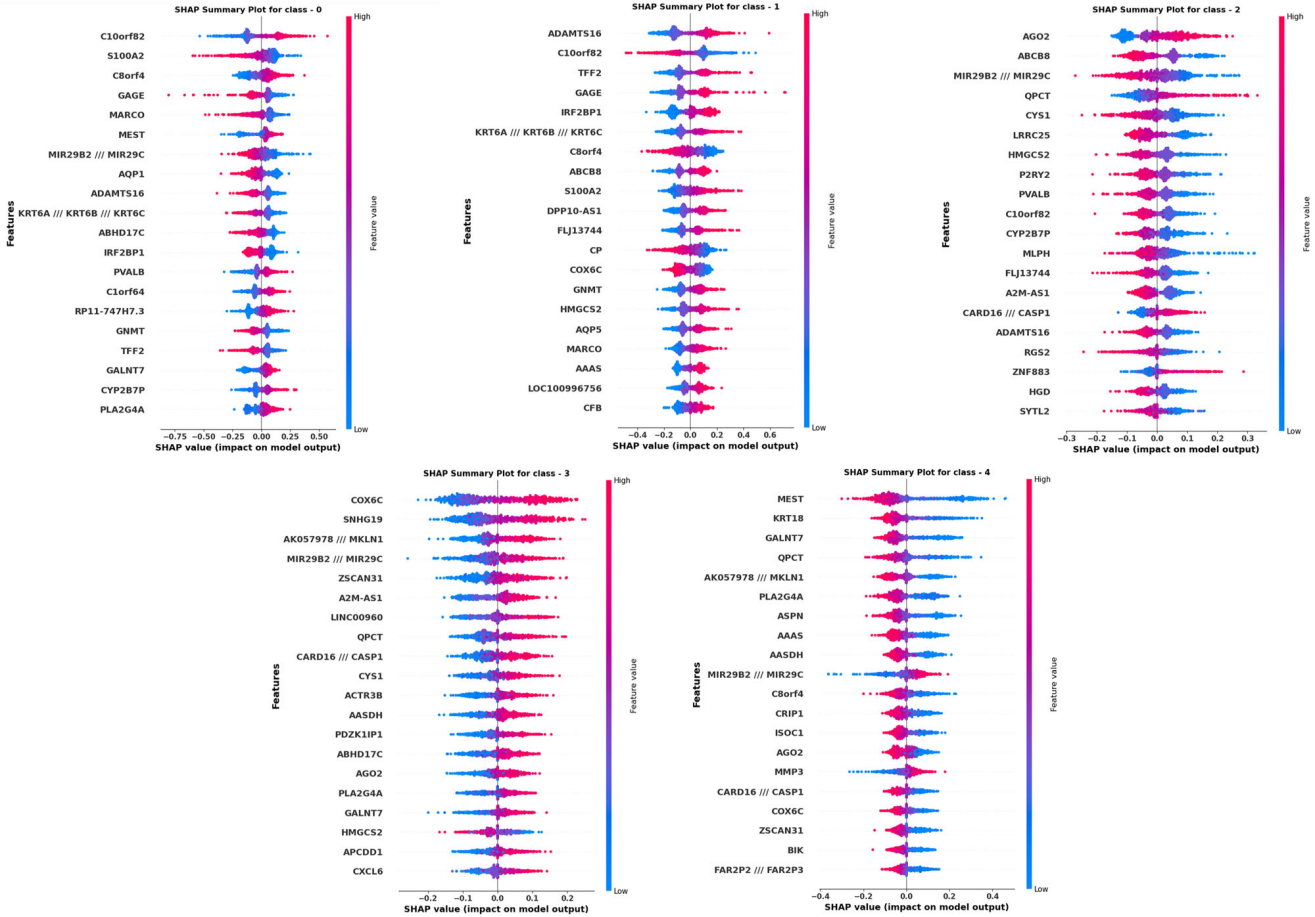
SHAP summary plots illustrating gene-level contributions to TNBC subtype and non-TNBC classification. SHAP summary plots are shown for each class (C0: non-TNBC; C1-C4: TNBC subtypes), where genes are ranked by mean absolute SHAP value, indicating their overall influence on model predictions; each point represents a sample, colored by normalized gene expression (red = high, blue = low), with the *x*-axis showing SHAP values that quantify the direction and magnitude of each gene’s contribution toward or against classification into the respective class.

SHAP explainability is employed for interpreting the ML models, specifically for gaining insights into the influence of genes on the classification of TNBC subtypes. As illustrated in Fig. 10, SHAP summary plots are generated for each subtype/cluster, including C0, representing the non-TNBC class.

Among the influential genes in determining non-TNBC samples (class 0), C10orf82, C8orf4, and CYP2B7P stand out. Since most of the points highlighted in red for these two genes are on the left side of the vertical axis, higher expression values of C10orf82 and S100A2 increase the likelihood of not classifying a sample as non-TNBC, suggesting a high negative correlation with class 0 (Non-TNBC). Conversely, since most of the points highlighted in red for this gene are on the right axis, higher expression values for C10orf82 gene would increase the chances of non-TNBC, suggesting a high positive correlation with class 0 (Fig. 10). However, S100A2 shows a high impact on negative correlation, thus indicating that lower expression of S100 calcium-binding protein A2 is high in non-TNBC class. For class 1, ADAMTS16, GAGE, C10orf82 and TFF2 are the top 4 most influential genes with ADAMTS16, GAGE and TFF2 having a high positive correlation/impact on class 1, while the opposite is suggested for C10orf82. Thus, a negative correlation of C10orf82 in both non-TNBC and C1 subtypes indicates mild similarity of some subtypes with other breast cancer types, which is similar to the results reported by other studies^26,44^. For class 2, AGO2 and QPCT have a high positive correlation with class 2. The recurrent prominence of C10orf82 across C0, C1 and, C2 subtypes suggests its potential role in breast cancer but lower impact on TNBC subtypes. For class 3, COX6C, SNHG19, and MKLN1 are some of the genes that have a high positive correlation with the class. For class 4, MEST, KRT18, and QPCT are some of the top genes that have a high negative correlation with the class.

### External validation and prognosis evaluation of biomarkers

The external validation results (Table 5), for models classifying between TNBC and Non-TNBC, confirm the robustness and generalizability of the developed models. Among the tested classifiers, the custom-built ensemble model has demonstrated the highest overall metrics, with accuracy (0.85), sensitivity (0.95), precision (0.71), and AUC-ROC (0.91), making it the most effective predictor. RF has also performed well, achieving an accuracy of 0.83 with an AUC-ROC of 0.90, though its precision (0.62) was lower than that of the ensemble model. In contrast, the DT model has exhibited the weakest performance, with an accuracy of 0.72 with an AUC-ROC of 0.80, indicating its limited ability to distinguish between classes.

**Table 5:** Performance summary of ML models after external validation

Model	Random forest	Decision tree	Support Vector machine	Gradient boosting	Custom built ensemble
Accuracy	0.83	0.72	0.75	0.80	0.85
Sensitivity	0.91	0.90	0.86	0.92	0.95
Specificity	0.75	0.7	0.73	0.82	0.81
Precision	0.62	0.54	0.62	0.64	0.71
Auc-roc	0.90	0.80	0.90	0.92	0.91

All the models have demonstrated high sensitivity, with the values reaching 0.95, indicating their strong ability to detect positive cases. However, the specificity has ranged from 0.7 to 0.8, suggesting that false positives remain an area for improvement. The custom-built ensemble model has balanced sensitivity (0.95) and specificity (0.81) effectively, making it the most reliable choice.

**Fig. 11:**
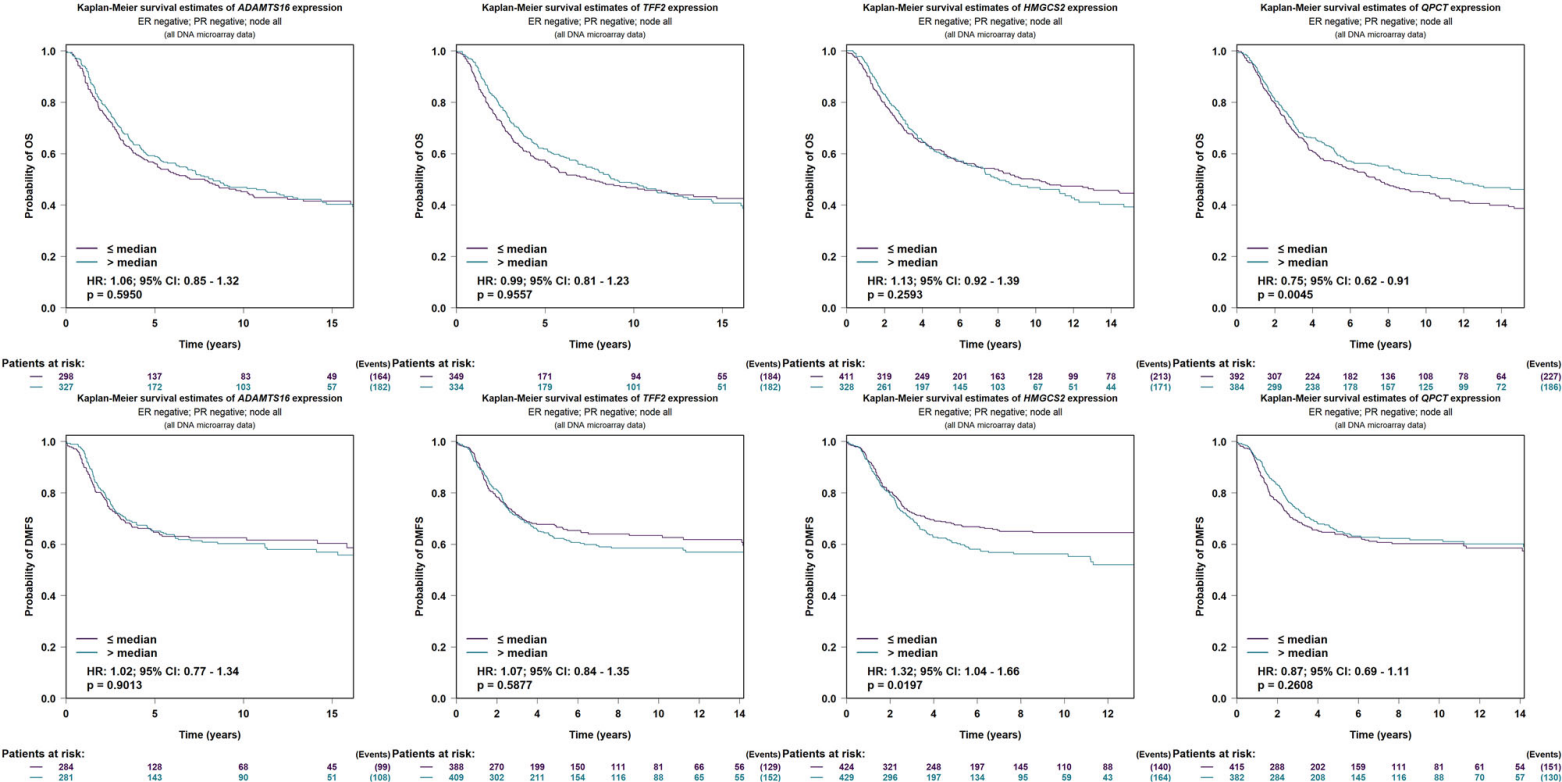
Prognostic evaluation of the identified genes of TNBC subtypes as per bc-GenExMiner in terms of overall survival (OS) and distant metastasis free survival (DMFS).

Prognostic evaluation of the genes of individual cluster are analyzed and reported in Fig. 11. It is noted that only QPCT shows statistically significant prognostic in overall survival. Similarly, only HMGCS2 shows significant distant metastasis free survival (DMFS) prognosis while other markers showed statistically poor correlation to DMFS.

## Discussion

The integration of multiple NCBI Gene Expression Omnibus (GEO) datasets allows the assembly of a large and diverse cohort of Triple Negative Breast Cancer samples. This solves the problem of having too few TNBC samples in resources like TCGA^13^. GEO remains a valuable and widely used repository for transcriptomic studies in TNBC, as demonstrated in previous investigations^13,15,45^. By aggregating several GEO datasets, this study enhances statistical robustness and improves the generalizability of subtype identification across heterogeneous populations. Unsupervised clustering of gene expression data further aids in delineating molecular heterogeneity within TNBC, a notion previously examined in the Lehman and Burstein classification systems^28,46^. While these studies are based on similar transcriptomic principles, differences in the number and definition of subtypes have been reported, with Lehman et al. later redefining TNBC into four subtypes by accounting for stromal and immune cell contributions^31^. The discovery of four stable subgroups in this study corroborates the viability of a streamlined and clinically useful classification system for diverse populations. Using K-means clustering, which is well-suited for gene expression data due to its reliance on mean expression patterns^47^, alongside hierarchical clustering, makes it even clearer that there are both unique and shared gene expression patterns among subtypes. This overlap highlights the inherent molecular complexity of TNBC, which is linked to aggressive clinical behavior and a significant risk of distant metastases within five years^48^. Such metastatic progression is driven by complex genetic and epigenetic alterations that promote tumor survival and dissemination^49^, emphasizing the need for improved subtype-specific biomarkers and predictive strategies to guide personalized therapeutic interventions.

Differential gene expression analysis is useful for more than only figuring out how different types of genes are transcribed. It is also a critical stage in deciding which features to utilize in machine learning applications, since having too many features might make models less stable and less useful. To avoid overfitting, it’s best to just look at genes that are statistically significant and biologically relevant. This keeps the ability to tell the difference between groups. Using Random Forest feature importance makes this approach even better by putting genes that help make predictions better at the top of the list. This accurately reflects nonlinear interactions prevalent in intricate gene expression data, enhancing the model’s generalisability^50^. This method works well with RFE, which iteratively improves the feature set to keep only the most important genes and cut down on noise and redundancy^40^. This strategic reduction in dimensionality is particularly important for pathway analysis because it focuses on genes that are biologically important and can tell the difference between different pathways. This makes enriched pathways easier to understand. For Triple Negative Breast Cancer, this kind of understanding is very important because the disease is very aggressive, and we need to know what molecular factors are behind subtype classification. Transparent feature selection and ranking facilitate the identification of candidate biomarkers that not only enhance accurate subtype prediction but also yield biologically significant insights that can guide personalised therapeutic strategies and clinical decision-making.

The enriched pathways identified among the TNBC subtypes further elucidate the molecular mechanisms driving tumor progression and therapeutic resistance. Some pathways in C1, like camera-type eye development and calcium ion transmembrane transport, may not seem like they have anything to do with breast cancer at first, but new research shows that genes linked to these pathways, like PAX6 and SOX9, may play a role in tumorigenesis, drug resistance, and possible therapeutic targeting^32,51–53^. The involvement of lateral and paraxial mesoderm pathways in C1 highlights the EMT as a critical mechanism that increases metastatic potential^33^. Subtypes C3 and C4 show an increase in pathways related to metastasis and therapy resistance, like controlling vascular permeability, peptide hormone release, and interferon type 1 signaling. These pathways may help tumor cells invade and avoid the immune system^34–36^. The dysregulation of autophagy, RNA metabolism, transcription, and signal transduction in C4 shows the intricacy of cellular adaptation mechanisms in TNBC. These findings draw attention to the fact that metastasis transpires among TNBC subtypes, with unique molecular pathways propelling disease advancement within each cluster, therefore requiring precise, subtype-specific therapeutic approaches customised to individual gene expression profiles.

Turning to the machine learning model development aspect of things, the choice of traditional machine learning models instead of deep learning models is warranted by the gene expression dataset’s moderate size and high dimensionality, as simpler models produce strong performance and improved interpretability while reducing overfitting^54^. FCA was useful for finding and getting rid of genes that are very similar to each other. This helps reduce multicollinearity and makes biological interpretation easier by showing co-expressed gene clusters that might have similar functions^39,55^. RFE improves the feature set even more by putting genes in order of how much they help model predictions^40,56,57^. This makes sure that the features chosen are both biologically relevant as well as useful for TNBC subtyping. Using DEG filtering, FCA, and RFE together not only makes the model more generalisable and less likely to overfit, but it also makes it better at picking up subtle gene expression patterns that are specific to certain subtypes. Ensemble models, especially the custom-built hard-voting classifier, use the strengths of each of the constituent classifiers to get consistently high accuracy, sensitivity, and AUC values across TNBC subtypes^42,43^. The variability in sensitivity observed among specific subtypes, including C2, points to the intrinsic molecular heterogeneity of TNBC^15,58,59^, indicating that more refined feature selection or alternative methodologies may further improve predictive performance.

SHAP-based explainability offers a unique aspect of interpretability ot the study. SHAP is a useful tool that allows us to see the contribution of individual genes to TNBC subtype classification, indicating both the extent and direction of their influence on model predictions. The analysis reveals subtype-specific gene signatures, notably ADAMTS16, GAUGE, and TFF2, which are significantly linked to C1. This suggests their potential roles in proteolysis, cell adhesion, and remodelling of the tumor microenvironment, paralleling functions attributed to other ADAM family members in cancer progression and immune modulation^60–62^. On the other hand, genes like C10orf82 show up a lot in different subtypes, which could mean that they play a bigger role in breast cancer biology but are not as good at telling TNBC apart from other types. This is in line with what previous studies have found^26,44^. Differences in gene correlations that are specific to each subtype, such as the opposite trends of ADAMTS16 between C1 and C2, show that TNBC is molecularly heterogeneous and that the models can pick up on subtle subtype-specific expression patterns. Furthermore, the participation of genes such as HMGCS2 in C3 and C2 points toward metabolic pathways, like ketogenesis, that may facilitate metastasis and disease advancement^63^.

Lastly, we end with external validation of our machine learning models. The high sensitivity across the models highlights their potential for identifying relevant biological signals. The superior performance of the ensemble model suggests that leveraging multiple classifiers helps mitigate weaknesses associated with individual models. These results validate the model’s reliability by ensuring independent evaluation^64^, underscoring the efficacy of the ensemble technique in optimising the balance of sensitivity, specificity, and total predictive power. It is important to note that even though this external validation does not involve classifying individual subtypes, it still does demonstrate that the trained model identifies biologically significant differences that extend beyond the internal dataset, thereby affirming its potential for translational application.

This study represents the first comprehensive application of explainable AI to simultaneously drive clustering, differential analysis, and mechanistic interpretation in TNBC. The findings lay a foundation for precision oncology by linking data-driven subtyping with actionable biological insights, ultimately supporting biomarker discovery and targeted treatment development in TNBC. We believe that the present study has pioneered the integration of conventional and interpretable ML models with SVM, tree-based, boosting and a custom-built voting ensemble classifier, to predict TNBC molecular subtypes. In this work, we have identified four common subtypes in different populations as a universal TNBC classification system irrespective of their demographic difference. Further exploration of the subtypes using pathway enrichment analysis has indicated the involvement of several significant pathways unique to each subtype. Our results have highlighted particularly the involvement of genes and pathways like GAGE, ADAMTS16, QPCT and HMGCS2 and how they are involved in subtype-selective vulnerabilities. In addition to this, we have comprehensively tested several ML models, including external validation, and identified the better performance of custom-built voting ensemble, SVM and RF models with high accuracy and higher recall. Unlike most cancer classification models that follow binary classifications like benign vs. malignant, this study has addressed multiclass classification. A key challenge in this type of multi-class classification lies in external validation. Although the model demonstrates strong performance on internal training and testing datasets, the external validation is currently restricted to distinguishing TNBC from non-TNBC because of the absence of externally available datasets with subtype-level annotations. Further analysis of the prognostic significance of the identified markers indicates the average OS and DFMS. For instance, QPCT expression has been identified to have a positive correlation to poor prognosis in patients treated with doxorubicin^65^. Similarly, it is established that HMCGS2 results in poor prognosis in ER+ patients^66^, indicating that this TNBC subtype prognosis can be similar to ER+ types. While HMCGS2 has been identified to be associated in tumor immune infiltration in renal carcinoma^67^, resulting in poor distant metastasis, its role in TNBC is elusive, demanding more research. However, genes like is TFF2 cannot be used as a prognostic marker which is in concordance with the results identified by Yi et al.^68^, who indicated that TFF1 plays a significant positive correlation to prognosis, unlike TFF2. However, a more validated gene signature panel is needed to further understand the role of these markers. We aim to focus on validating subtype-specific predictions using external cohorts or independent biological experiments to further strengthen translational relevance in the future. We believe that the present study not only illuminates the potential TNBC biomarkers for each subtype that surpass geographical constraints but also underscores the potential of ensemble classifiers in the realm of genomics research.

## Methodology

The identification of distinct subtypes of cancer can provide insights into the genetic, epigenetic, and transcriptional profiles of cancer. This facilitates biomarker discovery for early diagnosis. Through the integration and analysis of heterogeneous gene expression data, clustering methodologies unveil discrete subtypes, paving the way for the advancement of refined and personalized treatment strategies^28,46^. Subsequently, ML can be adapted to classify the identified subtypes, enabling the creation of robust models for class prediction and patient stratification^69^.

### Dataset design and pre-processing

As an initial step towards TNBC subtyping, the acquisition of a comprehensive dataset is of paramount importance^23^. Considering the abundant availability and diversity of GEO datasets, we choose to work with NCBI GEO omnibus datasets^70^. It is crucial to acknowledge that the NCBI database encompasses a wide range of data derived from various experimental platforms. Choice of uniform datasets could result in batch effects, which could impact the accuracy of downstream analyzes and ML models^71^. Hence, we choose to amalgamate samples from the same [HG-U133_Plus_2] Affymetrix Human Genome U133 Plus 2.0 Array platform. The series matrix files from six datasets, including GSE76275, GSE18864, GSE58812, GSE65194, GSE83937, and GSE95700 are combined in this study. These datasets are strategically chosen to enable the identification of a universal gene signature, irrespective of demographic differences. To further avoid batch effect, we use the pycombat package, which implements the ComBat algorithm, a well-established empirical Bayes method for batch effect correction^72^. Batch correction ensures the removal of unwanted technical variation while preserving true biological differences. This further strengthens the reliability of downstream analyzes, ensuring that findings are driven by meaningful biological signals rather than confounding batch effects^72,73^.

Initial preprocessing is done in the R console, including data curation and cleaning. Processing is performed on the input data to counter the missing values, inconsistent and erroneous entries, and duplicate genes, enhancing the dataset’s reliability. The duplication of genes is managed by calculating the mean expression values to mitigate any potential effects of cross-hybridization. This is certainly a concern in microarray experiments that may arise due to probe sequence similarities, causing the same gene symbol to appear in multiple locations^28^. Subsequently, each dataset is subjected to quantile normalization and a log-base-2 transformation, ensuring uniformity among the data ranges. After data curation and cleaning, a combined dataset of 807 samples with 565 TNBC and 242 non-TNBC samples has been achieved.

Before performing any clustering, differential expression analysis (DEG), feature correlation filtering, RFE, or model training, we first split the complete dataset into an 80/20 train-test partition. The preprocessing steps, including clustering, DEG selection, correlation-based feature removal, RFE-based feature selection, and model development, are performed exclusively on the training set to avoid information leakage. The held-out test set is kept completely separate during the entire pipeline and is used only once at the end to evaluate model performance. This ensures that the reported results reflect the model’s true generalizability and real-world performance, rather than optimistic estimates influenced by training data.

#### TNBC clustering

We perform clustering on the training portion of the combined dataset using two conventional clustering analysis; K-means and hierarchical clustering are performed to derive a universal classification system despite the demographic differences; this is an initial step towards ML-based classification. A within-cluster sum of squares (WCSS) integrates seamlessly with K-means clustering and rarely demands any additional resources. The value of *K* was then chosen by comparing clustering results and the corresponding WCSS for various *K* values using the Elbow method. WCSS is given by: $WCSS={\sum }_{i=n}{({X}_{i}-{Y}_{i})}^{2}$, where *Y*_*i*_ is the centroid for sample *X*_*i*_ and *n* is the total number of samples.

Consequently, K-means clustering is performed on the TNBC training dataset; it utilizes the results to assign subtypes to individual samples. Well-defined clusters are labeled accordingly as C1, C2, C3, and so on. A set of samples that belong to non-TNBC is assigned the label C0. Simultaneously, hierarchical clustering is carried out on the combined dataset to validate the obtained clusters. This sequential clustering progression is visually represented by a hierarchical binary tree or dendrogram. By selectively cutting this dendrogram to a specified hierarchical level, one could derive a desired number of clusters. A similar labeling system is followed for easier interpretation.

#### Differential expression analysis

After clustering analysis, we identify the patterns in gene expression that are specific or unique to each cluster. This serves as an effective feature selection method as it could help identify the most informative genes determining the difference in the subtypes. *R* limma package has been used to identify DEGs using the statistical criteria log fold change >2 and adjusted (FDR or Benjamini–Hochberg correction) *p* < 0.05^13^. After DEG analysis, we find 944 DEGs across the four subtypes, with some upregulated and some downregulated. Furthermore, DEG analysis for each cluster is performed to identify the unique DEGs exhibited within each cluster/subtype. The unique DEGs for each subtype are determined by comparing the expression levels of the genes for that subtype as compared to the other subtypes.

#### Pathway enrichment analysis

A sophisticated feature selection process is pivotal to unraveling the genetic determinants that underlie the subtypes of TNBC and selecting the most influential genes for pathway analyzes by isolating the most impactful genes. Random Forest (RF) ranks features based on their importance scores, making it well-suited for high dimensional data like gene expression^50^. Unlike Lasso, which enforces sparsity, or Ridge, which mitigates multicollinearity, RF captures complex gene interactions without assuming linearity. Its ability to handle noisy data and rank informative genes ensures a robust selection process, aiding in identifying key predictors for TNBC classification.

Pathway enrichment analysis of the unique DEGs for each cluster is performed using the Metascape tool^74^. We perform enrichment analysis using GO Biological Processes, Reactome Gene Sets, Canonical Pathways, and CORUM^75^. The analysis is completed with *p*-value of <0.01, a minimum count of 3, and an enrichment factor of >2.0.

### Study description

This study delves into gene expression patterns to gain insights into the subtypes identified through prior clustering analysis. Initially, the training dataset is pre-processed by applying feature scaling to the missing values and eliminating erroneous entries. We normalize the data to homogenize using the standard scaler^76^ facilitating the alignment of diverse features onto a commensurate scale. This entails the centralization of all feature columns, aligning their means to zero, and standardizing their deviations to one, thereby retaining crucial information on outliers.

The next step involves a quantitative assessment of the linear relationships between pairs of features by employing the PCC. To scrutinize the relationship among the different gene expression features, a comprehensive FCA is adopted^77^. This is achieved by using the PCC filter-based approach to limit the number of redundant features.

1 $$P=\frac{{\sum }_{k=1}^{K}({g}_{ik}-{ {\bar{g}} }_{i})({g}_{jk}-{ {\bar{g}} }_{j})}{\sqrt{{\sum }_{k=1}^{K}{({g}_{ik}-{ {\bar{g}} }_{i})}^{2}}\sqrt{{\sum }_{k=1}^{K}{({g}_{jk}-{ {\bar{g}} }_{j})}^{2}}}$$

FCA is performed on the DEG obtained from Limma DEG analysis. Highly correlated features (80%) are removed to avoid multicollinearity and overfitting in the ML models.

Before model development, RFE is also employed to further refine the feature set. RFE iteratively removes the least important features based on model performance, enhancing predictive accuracy and reducing overfitting^40^.

The selection of parameters has a considerable impact on the accuracy of ML models. Simultaneously, a biased dataset split might fail to effectively classify the characteristics into separate subtypes^78^. Hence, to maintain the integrity of the analysis, we use stratification in the training process. This strategic separation mitigates the risk of data leakage during subsequent steps as well as prevents sampling imbalance, providing a robust bias-free foundation for model evaluation^79^. In the initial studies, four ML models are evaluated using the gene expression data without any feature selection process or upsampling to provide baseline performance. To train the models effectively, we apply fivefold stratified cross-validation on the training set, training and validating the models across multiple folds, improving robustness. The final selected model is then evaluated on the held-out 20% test set, which has not been used at any stage of training or feature selection.

In the next stage, gene expression data are used after DEG analysis, FCA, and RFE to study the model performance.

#### ML models and evaluation metrics

Four different ML modes are employed to classify the TNBC samples into clusters. The standard ML models like SVM, RF, DT, and XGBoost are chosen owing to their simplicity and interpretability^80^. In addition to these conventional ML models, a voting-based custom-built ensemble model is used to combine the decisive power of the models in the classification task. Among the different ML models, these four have been chosen to compare the differences in their underlying architecture in analyzing gene expression. For instance, SVM identifies an optimal plane within the high-dimensional feature space to maximize the margin between instances of two classes. In contrast, tree-based ML models utilize features to create decision rules at the nodes and provide classification outcomes at the leaves, either as individual trees (DT) or as ensemble (RF).

The models are comprehensively evaluated against several metrics such as accuracy, sensitivity, specificity, precision, AUC-ROC, and F1.

#### SHapley Additive exPlanations (SHAP) Analysis for XAI

In the final phase of the study, SHAP analysis is incorporated to enhance the interpretability of the ML models^81^. The primary objective is to identify the most influential genes contributing to the classification of TNBC subtypes. The SHAP analysis involves computing Shapley values for each gene, allowing to discern the marginal contribution of genes to the differences between actual predictions and expected predictions.

#### External validation

The held-out test set (20% of data) provides a strong form of internal validation, since it remains completely isolated from the clustering, DEG selection, feature selection, and model training processes. In addition to this internal validation, we also looked at how well the results might be applied to other situations by utilizing an external validation dataset. We extract the dataset, GSE43358, from the same platform in GEO, for the purpose of external validation. The goal was made simpler by only looking at TNBC vs. non-TNBC (with non-TNBC being subtype 0 and all other subtypes being TNBC).

To further analyze the prognostic significance of the genes, bc-GenExMiner v3.0 is used. This platform enables prognosis evaluation of genes based on transcriptomic and pathological data from both microarray and RNAseq data^82^. The prognostic significance is studied for an identified set of genes with ER, PR negative, HER2 negative statuses. All node status is included to identify both overall survival (OS) ad DMFS.

### Implementation details

We utilize a combination of R and Python programming languages to achieve a comprehensive analysis. In R, the GEOquery library is employed for the collection of series matrix files from the GEO, and the limma package facilitates the differential gene expression analysis. Visualization of the results is accomplished using the ggplot2 library to generate informative volcano plots.

Turning to Python, hierarchical clustering and dendrogram plotting are executed using the scipy library, providing insights into gene expression patterns. Machine learning tasks, including K-means clustering and loading of classification models, are performed using the scikit-learn library. Further steps, such as scaling and feature selection, are also implemented with scikit-learn. The shap library is used for shap analysis. Additionally, the performance metrics for classification models are calculated and loaded using scikit-learn to ensure a robust evaluation of model effectiveness.

The training of ML models and the computations are carried out on a HP Z8 workstation with 128 GB of RAM and a 64-core Intel® Xeon(R) Silver 4216 CPU with a 2.10 GHz base clock.

## Data Availability

The public gene expression data on TNC were retrieved from the Gene Expression Omnibus (GEO) database. The transcriptomic datasets supporting the findings of this study are publicly available in the GEO repository under the following accession numbers: GSE76275, GSE18864, GSE58812, GSE65194, GSE83937, and GSE95700.

## Code availability

The custom code and scripts developed for this study-including dataset preparation, subtype clustering, differential expression analysis, and machine learning model development-are publicly available on GitHub at https://github.com/syedmohs/Triple-negative-breast-cancer/tree/main. The computational pipeline is implemented using Python (v3.9.12) and R (v4.2.1). Key software dependencies include:Python Libraries: scikit-learn (StandardScaler, K-means, RFE), pandas, numpy, shap (v0.41.0), and Jupyter Notebook.R Packages: limma (v3.52.2) for differential expression analysis.Specific parameters used in the analysis include K-means clustering with *k* = 4 (assigning subtypes C0-C4), and differential expression thresholds of ∣*l**o**g**F**C*∣ > 2 with an adjusted *p*-value < 0.05. For machine learning, feature selection was performed by removing variables with a correlation >0.80 followed by Recursive Feature Elimination (RFE) to identify the top 50 features. All models were validated using stratified 5-fold cross-validation. A detailed run-sheet describing the execution order of the scripts (Dataset preparation.ipynb, Subtyping.ipynb, DEG.R, and Model Development.ipynb) is provided in the repository.

## References

[1] Sung, H. et al. Global cancer statistics 2020: Globocan estimates of incidence and mortality worldwide for 36 cancers in 185 countries. *CA Cancer J. Clin.* **71**, 209–249 (2021). 33538338 10.3322/caac.21660

[2] Wu, S. et al. A novel axis of circkif4a-mir-637-stat3 promotes brain metastasis in triple-negative breast cancer. *Cancer Lett.* **581**, 216508 (2024). 38029538 10.1016/j.canlet.2023.216508

[3] Lui, J. W. et al. Trps1 is a promising marker for all subtypes of breast cancer. *Histopathology* **84**, 822–836 (2024). 38173281 10.1111/his.15126

[4] Ge, L.-P. et al. Znf689 deficiency promotes intratumor heterogeneity and immunotherapy resistance in triple-negative breast cancer. *Cell Res.* **34**, 58–75 (2024). 38168642 10.1038/s41422-023-00909-w PMC10770380

[5] Liu, S., Zhang, Y. & Shang, X. Glassonet: Identifying discriminative gene sets among molecular subtypes of breast cancer. *IEEE/ACM Trans. Comput. Biol. Bioinform.* **20**, 1905–1916 (2023). 36346852 10.1109/TCBB.2022.3220623

[6] Hassan, M. et al. Innovations in genomics and big data analytics for personalized medicine and health care: a review. *Int. J. Mol. Sci.* **23**, 4645 (2022). 35563034 10.3390/ijms23094645 PMC9104788

[7] Schmid, P. et al. First-line ipatasertib, atezolizumab, and taxane triplet for metastatic triple-negative breast cancer: clinical and biomarker results. *Clin. Cancer Res.* **30**, 767–778 (2024). 38060199 10.1158/1078-0432.CCR-23-2084 PMC10870115

[8] Lehmann, B. D. & Pietenpol, J. A. Identification and use of biomarkers in treatment strategies for triple-negative breast cancer subtypes. *J. Pathol.* **232**, 142–50 (2014). 24114677 10.1002/path.4280 PMC4090031

[9] Prat, A. et al. Clinical implications of the intrinsic molecular subtypes of breast cancer. *Breast* **24**, S26–S35 (2015). 26253814 10.1016/j.breast.2015.07.008

[10] McCart Reed, A. E., Kalita-De Croft, P., Kutasovic, J. R., Saunus, J. M. & Lakhani, S. R. Recent advances in breast cancer research impacting clinical diagnostic practice. *J. Pathol.* **247**, 552–562 (2019). 30426489 10.1002/path.5199

[11] Mendonca-Neto, R. et al. A gene selection method based on outliers for breast cancer subtype classification. *IEEE/ACM Trans. Comput. Biol. Bioinform.* **19**, 2547–2559 (2021). 10.1109/TCBB.2021.3132339 34860652

[12] Chen, W. et al. Risk prediction of pancreatic cancer in patients with recent-onset hyperglycemia: a machine-learning approach. *J. Clin. Gastroenterol.* **57**, 103–110 (2023). 35470312 10.1097/MCG.0000000000001710 PMC9585151

[13] Thalor, A., Joon, H. K., Singh, G., Roy, S. & Gupta, D. Machine learning assisted analysis of breast cancer gene expression profiles reveals novel potential prognostic biomarkers for triple-negative breast cancer. *Comput. Struct. Biotechnol. J.* **20**, 1618–1631 (2022). 35465161 10.1016/j.csbj.2022.03.019 PMC9014315

[14] DeRisi, J. et al. Use of a cdna microarray to analyse gene expression. *Nat. Genet.* **14**, 457–460 (1996). 8944026 10.1038/ng1296-457

[15] Bissanum, R., Chaichulee, S., Kamolphiwong, R., Navakanitworakul, R. & Kanokwiroon, K. Molecular classification models for triple negative breast cancer subtype using machine learning. *J. Person. Med.* **11**, 881 (2021). 10.3390/jpm11090881 PMC8472680 34575658

[16] Zanella, L., Facco, P., Bezzo, F. & Cimetta, E. Feature selection and molecular classification of cancer phenotypes: a comparative study. *Int. J. Mol. Sci.* **23**, 9087 (2022). 36012350 10.3390/ijms23169087 PMC9408964

[17] Nasir, I. M. et al. Pearson correlation-based feature selection for document classification using balanced training. *Sensors* **20**, 6793 (2020). 33261136 10.3390/s20236793 PMC7730850

[18] Shukla, A. K., Singh, P. & Vardhan, M. A hybrid gene selection method for microarray recognition. *Biocybern. Biomed. Eng.* **38**, 975–991 (2018).

[19] Park, B., Lee, W. & Han, K. A new approach to deriving prognostic gene pairs from cancer patient-specific gene correlation networks. *IEEE/ACM Trans. Comput. Biol. Bioinform.* **19**, 1267–1276 (2020). 10.1109/TCBB.2020.3017209 32809942

[20] Hases, L. et al. The importance of sex in the discovery of colorectal cancer prognostic biomarkers. *Int. J. Mol. Sci.* **22**, 1354 (2021). 33572952 10.3390/ijms22031354 PMC7866425

[21] Dwivedi, A. K. Artificial neural network model for effective cancer classification using microarray gene expression data. *Neural Comput. Appl.* **29**, 1545–1554 (2018).

[22] Mazlan, A. U. et al. A review on recent progress in machine learning and deep learning methods for cancer classification on gene expression data. *Processes* **9**, 1466 (2021).

[23] Mostavi, M., Chiu, Y.-C., Huang, Y. & Chen, Y. Convolutional neural network models for cancer type prediction based on gene expression. *BMC Med. Genom.* **13**, 1–13 (2020). 10.1186/s12920-020-0677-2 PMC7119277 32241303

[24] Lee, S., Lim, S., Lee, T., Sung, I. & Kim, S. Cancer subtype classification and modeling by pathway attention and propagation. *Bioinformatics* **36**, 3818–3824 (2020). 32207514 10.1093/bioinformatics/btaa203

[25] Yin, L., Duan, J.-J., Bian, X.-W. & Yu, S. -c Triple-negative breast cancer molecular subtyping and treatment progress. *Breast Cancer Res.* **22**, 1–13 (2020). 10.1186/s13058-020-01296-5 PMC7285581 32517735

[26] Ensenyat-Mendez, M. et al. Current triple-negative breast cancer subtypes: dissecting the most aggressive form of breast cancer. *Front. Oncol.* **11**, 681476 (2021). 34221999 10.3389/fonc.2021.681476 PMC8242253

[27] Liu, J., Su, R., Zhang, J. & Wei, L. Classification and gene selection of triple-negative breast cancer subtype embedding gene connectivity matrix in deep neural network. *Brief. Bioinform.* **22**, bbaa395 (2021). 33415328 10.1093/bib/bbaa395

[28] Burstein, M. D. et al. Comprehensive genomic analysis identifies novel subtypes and targets of triple-negative breast cancer. *Clin. Cancer Res.* **21**, 1688–98 (2015). 25208879 10.1158/1078-0432.CCR-14-0432 PMC4362882

[29] Sehhati, M., Tabatabaiefar, M. A., Gholami, A. H. & Sattari, M. Using classification and k-means methods to predict breast cancer recurrence in gene expression data. *J. Med. Signals Sens.* **12**, 122 (2022). 35755980 10.4103/jmss.jmss_117_21 PMC9215834

[30] Kakushadze, Z. & Yu, W. * k-means and cluster models for cancer signatures. *Biomol. Detect. Quantif.* **13**, 7–31 (2017). 29021969 10.1016/j.bdq.2017.07.001 PMC5634820

[31] Lehmann, B. D. et al. Refinement of triple-negative breast cancer molecular subtypes: Implications for neoadjuvant chemotherapy selection. *PLoS ONE* **11**, e0157368 (2016). 27310713 10.1371/journal.pone.0157368 PMC4911051

[32] Guo, X.-F., Wang, L.-L., Zheng, F.-M. & Li, H.-P. Pax6 enhances nanog expression by inhibiting notch signaling to promote malignant properties in small cell lung cancer cells. *Heliyon* **11**, e41795 (2025). 39885876 10.1016/j.heliyon.2025.e41795 PMC11780942

[33] Newton, A. H. & Smith, C. A. Resolving the mechanisms underlying epithelial-to-mesenchymal transition of the lateral plate mesoderm. *Genesis* **62**, e23531 (2024). 37443419 10.1002/dvg.23531

[34] Tomita, T., Kato, M. & Hiratsuka, S. Regulation of vascular permeability in cancer metastasis. *Cancer Sci.* **112**, 2966–2974 (2021). 33966313 10.1111/cas.14942 PMC8353911

[35] Martin, J. C. et al. Aryl hydrocarbon receptor suppresses sting-mediated type I IFN expression in triple-negative breast cancer. *Sci. Rep.* **14**, 5731 (2024). 38459088 10.1038/s41598-024-54732-3 PMC10923803

[36] Zimmerli, D. et al. Myc promotes immune-suppression in triple-negative breast cancer via inhibition of interferon signaling. *Nat. Commun.* **13**, 6579 (2022). 36323660 10.1038/s41467-022-34000-6 PMC9630413

[37] Mao, Y., Zhou, X., Pi, D., Sun, Y. & Wong, S. T. Multiclass cancer classification by using fuzzy support vector machine and binary decision tree with gene selection. *J. Biomed. Biotechnol.* **2005**, 160 (2005). 16046822 10.1155/JBB.2005.160 PMC1184049

[38] Anand, A. & Suganthan, P. N. Multiclass cancer classification by support vector machines with class-wise optimized genes and probability estimates. *J. Theor. Biol.* **259**, 533–540 (2009). 19406131 10.1016/j.jtbi.2009.04.013

[39] Miller, H. E. & Bishop, A. J. Correlation analyzer: functional predictions from gene co-expression correlations. *BMC Bioinform.* **22**, 1–19 (2021). 10.1186/s12859-021-04130-7 PMC8056587 33879054

[40] Zeng, L. & Chen, Z. Screening of genes characteristic of pancreatic cancer by lasso regression combined with support vector machine and recursive feature elimination, and immune correlation analysis. *J. Int. Med. Res.* **52**, 03000605241233160 (2024). 38456653 10.1177/03000605241233160 PMC10924566

[41] Shen, J. et al. Deep learning approach for cancer subtype classification using high-dimensional gene expression data. *BMC Bioinform.* **23**, 1–17 (2022). 10.1186/s12859-022-04980-9 PMC9575247 36253710

[42] Ding, S., Zheng, J. & Jia, C. Deepmens: an ensemble model for predicting sgrna on-target activity based on multiple features. *Brief. Funct. Genom.* **24**, elae043 (2025). 10.1093/bfgp/elae043 PMC11735754 39528429

[43] Maigari, A., Zainol, Z. & Xinying, C. Multi-modal stacked ensemble model for breast cancer prognosis prediction. *Stat. Optim. Inf. Comput.* **13**, 1013–1034 (2025).

[44] Wang, D.-Y., Jiang, Z., Ben-David, Y., Woodgett, J. R. & Zacksenhaus, E. Molecular stratification within triple-negative breast cancer subtypes. *Sci. Rep.* **9**, 19107 (2019). 31836816 10.1038/s41598-019-55710-w PMC6911070

[45] Bolón-Canedo, V., Sánchez-Maroño, N., Alonso-Betanzos, A., Benítez, J. M. & Herrera, F. A review of microarray datasets and applied feature selection methods. *Inf. Sci.* **282**, 111–135 (2014).

[46] Lehmann, B. D. et al. Identification of human triple-negative breast cancer subtypes and preclinical models for selection of targeted therapies. *J. Clin. Invest.* **121**, 2750–67 (2011). 21633166 10.1172/JCI45014 PMC3127435

[47] Jiang, W., Joehanes, R., Levy, D., O’Connor, G. T. & Dupuis, J. Assisted clustering of gene expression data using regulatory data from partially overlapping sets of individuals. *BMC Genom.* **23**, 819 (2022). 10.1186/s12864-022-09026-1 PMC9734806 36496393

[48] Al-Mahmood, S., Sapiezynski, J., Garbuzenko, O. B. & Minko, T. Metastatic and triple-negative breast cancer: challenges and treatment options. *Drug Deliv. Transl. Res.* **8**, 1483–1507 (2018). 29978332 10.1007/s13346-018-0551-3 PMC6133085

[49] Valastyan, S. & Weinberg, R. A. Tumor metastasis: molecular insights and evolving paradigms. *Cell* **147**, 275–292 (2011). 22000009 10.1016/j.cell.2011.09.024 PMC3261217

[50] Li, J. et al. Identification and validation of cuproptosis-related genes for diagnosis and therapy in nonalcoholic fatty liver disease. *Mol. Cell. Biochem.* **480**, 473–489 (2025). 38512536 10.1007/s11010-024-04957-7

[51] Jiang, J. et al. Sox on tumors, a comfort or a constraint? *Cell Death Discov.* **10**, 67 (2024). 38331879 10.1038/s41420-024-01834-6 PMC10853543

[52] Jin, M., Gao, D., Wang, R., Sik, A. & Liu, K. Possible involvement of tgf-*β*-smad-mediated epithelial-mesenchymal transition in pro-metastatic property of Pax6. *Oncol. Rep.* **44**, 555–564 (2020). 32627030 10.3892/or.2020.7644 PMC7336511

[53] Tripathi, S. K., Sahoo, R. K. & Biswal, B. K. Sox9 as an emerging target for anticancer drugs and a prognostic biomarker for cancer drug resistance. *Drug Discov. Today* **27**, 2541–2550 (2022). 35636723 10.1016/j.drudis.2022.05.022

[54] Wani, N. A., Kumar, R. & Bedi, J. Deepxplainer: an interpretable deep learning based approach for lung cancer detection using explainable artificial intelligence. *Comput. Methods Prog. Biomed.* **243**, 107879 (2024). 10.1016/j.cmpb.2023.107879 37897989

[55] Roy, S., Kumar, R., Mittal, V. & Gupta, D. Classification models for invasive ductal carcinoma progression, based on gene expression data-trained supervised machine learning. *Sci. Rep.* **10**, 4113 (2020). 32139710 10.1038/s41598-020-60740-w PMC7057992

[56] Wahid, A. et al. Feature selection and classification for gene expression data using novel correlation based overlapping score method via chou’s 5-steps rule. *Chemomet. Intell. Lab. Syst.* **199**, 103958 (2020).

[57] Seetharaman, A. & Sundersingh, A. C. Gene selection and classification using correlation feature selection based binary bat algorithm with greedy crossover. *Concurr. Comput. Pract. Exper.* **34**, e6718 (2022).

[58] Turova, P. et al. The breast cancer classifier refines molecular breast cancer classification to delineate the her2-low subtype. *NPJ Breast Cancer* **11**, 19 (2025). 39979291 10.1038/s41523-025-00723-0 PMC11842814

[59] Zhang, Y. et al. Tnbc molecular subtypes and potential detection targets for biological therapy indications. *Carcinogenesis* **46**, bgaf006 (2025). 39977309 10.1093/carcin/bgaf006

[60] Navasatli, S. A. et al. New insight into the role of the Adam protease family in breast carcinoma progression. *Heliyon* **10**, e24805 (2024). 38317965 10.1016/j.heliyon.2024.e24805 PMC10839977

[61] Xiao, L. et al. Adamts16 drives epithelial-mesenchymal transition and metastasis through a feedback loop upon tgf-*β*1 activation in lung adenocarcinoma. *Cell Death Dis.* **15**, 837 (2024). 39551781 10.1038/s41419-024-07226-z PMC11570625

[62] Liang, L., Zhu, J. -h, Chen, G., Qin, X. -g & Chen, J. -q Prognostic values for the mrna expression of the Adamts family of genes in gastric cancer. *J. Oncol.* **2020**, 9431560 (2020). 32884571 10.1155/2020/9431560 PMC7455834

[63] Jiang, H. et al. Ketogenesis promotes triple-negative breast cancer metastasis via calpastatin *β*-hydroxybutyrylation. *Lipids Health Dis.* **23**, 371 (2024). 39533307 10.1186/s12944-024-02364-x PMC11555945

[64] Yao, F., Luo, J., Zhou, Q., Wang, L. & He, Z. Development and validation of a machine learning-based prediction model for hepatorenal syndrome in liver cirrhosis patients using MIMIC-IV and eICU databases. *Sci. Rep.* **15**, 2743 (2025). 39837961 10.1038/s41598-025-86674-9 PMC11751441

[65] Xu, B. et al. Role of glutaminyl-peptide cyclotransferase in breast cancer doxorubicin sensitivity. *Cancer Biol. Ther.* **25**, 2321767 (2024). 38417050 10.1080/15384047.2024.2321767 PMC10903679

[66] Hwang, S. et al. Targeting hmg-coa synthase 2 suppresses tamoxifen-resistant breast cancer growth by augmenting mitochondrial oxidative stress-mediated cell death. *Life Sci.* **328**, 121827 (2023). 37276910 10.1016/j.lfs.2023.121827

[67] Mao, H. et al. Hmgcs2 serves as a potential biomarker for inhibition of renal clear cell carcinoma growth. *Sci. Rep.* **13**, 14629 (2023). 37670031 10.1038/s41598-023-41343-7 PMC10480187

[68] Yi, J. et al. Trefoil factor 1 (tff1) is a potential prognostic biomarker with functional significance in breast cancers. *Biomed. Pharmacother.* **124**, 109827 (2020). 31986408 10.1016/j.biopha.2020.109827

[69] Ding, R. et al. Identification of breast cancer subtypes by integrating genomic analysis with the immune microenvironment. *ACS Omega* **8**, 12217–12231 (2023). 37033796 10.1021/acsomega.2c08227 PMC10077467

[70] Edgar, R., Domrachev, M. & Lash, A. E. Gene expression omnibus: NCBI gene expression and hybridization array data repository. *Nucleic Acids Res.* **30**, 207–10 (2002). 11752295 10.1093/nar/30.1.207 PMC99122

[71] Papiez, A., Marczyk, M., Polanska, J. & Polanski, A. Batchi: Batch effect identification in high-throughput screening data using a dynamic programming algorithm. *Bioinformatics* **35**, 1885–1892 (2019). 30357412 10.1093/bioinformatics/bty900 PMC6546123

[72] Kotlov, N. et al. Procrustes is a machine-learning approach that removes cross-platform batch effects from clinical rna sequencing data. *Commun. Biol.* **7**, 392 (2024). 38555407 10.1038/s42003-024-06020-z PMC10981711

[73] Yu, Y., Mai, Y., Zheng, Y. & Shi, L. Assessing and mitigating batch effects in large-scale omics studies. *Genome Biol.* **25**, 254 (2024). 39363244 10.1186/s13059-024-03401-9 PMC11447944

[74] Zhou, Y. et al. Metascape provides a biologist-oriented resource for the analysis of systems-level datasets. *Nat. Commun.* **10**, 1523 (2019). 30944313 10.1038/s41467-019-09234-6 PMC6447622

[75] Jassal, B. et al. The reactome pathway knowledgebase. *Nucleic Acids Res.* **48**, D498–D503 (2020). 31691815 10.1093/nar/gkz1031 PMC7145712

[76] Prado-Vázquez, G. et al. A novel approach to triple-negative breast cancer molecular classification reveals a luminal immune-positive subgroup with good prognoses. *Sci. Rep.* **9**, 1538 (2019). 30733547 10.1038/s41598-018-38364-y PMC6367406

[77] Hou, J. et al. Distance correlation application to gene co-expression network analysis. *BMC Bioinform.* **23**, 1–24 (2022). 10.1186/s12859-022-04609-x PMC8862277 35193539

[78] Ansari, M. Y., Chandrasekar, V., Singh, A. V. & Dakua, S. P. Re-routing drugs to blood brain barrier: A comprehensive analysis of machine learning approaches with fingerprint amalgamation and data balancing. *IEEE Access* **11**, 9890–9906 (2022).

[79] Ünalan, S., Günay, O., Akkurt, I., Gunoglu, K. & Tekin, H. A comparative study on breast cancer classification with stratified shuffle split and k-fold cross validation via ensembled machine learning. *J. Radiat. Res. Appl. Sci.* **17**, 101080 (2024).

[80] Roy, S., Singh, J. & Ray, S. S. Weighted combination of łukasiewicz implication and Jaccard similarity in hybrid ensemble learning framework (wcljhelf) for gene selection. *Comput. Biol. Med.* **170**, 107981 (2024). 38262204 10.1016/j.compbiomed.2024.107981

[81] Khater, T., Ansari, S., Mahmoud, S., Hussain, A. & Tawfik, H. Skin cancer classification using explainable artificial intelligence on pre-extracted image features. *Intell. Syst. Appl.* **20**, 200275 (2023).

[82] Jézéquel, P. et al. bc-genexminer 4.5: new mining module computes breast cancer differential gene expression analyses. *Database* **2021**, baab007 (2021). 33599248 10.1093/database/baab007 PMC7904047

[83] Eo, H.-S., Heo, J. Y., Choi, Y., Hwang, Y. & Choi, H.-S. A pathway-based classification of breast cancer integrating data on differentially expressed genes, copy number variations and microRNA target genes. *Mol. Cells* **34**, 393–398 (2012). 22983731 10.1007/s10059-012-0177-0 PMC3887768

[84] Gopal, V. N., Al-Turjman, F., Kumar, R., Anand, L. & Rajesh, M. Feature selection and classification in breast cancer prediction using iot and machine learning. *Measurement* **178**, 109442 (2021).

[85] Huang, H. H., Liu, X. Y. & Liang, Y. Feature selection and cancer classification via sparse logistic regression with the hybrid l1/2 +2 regularization. *PLoS ONE* **11**, e0149675 (2016). 27136190 10.1371/journal.pone.0149675 PMC4852916

[86] Akpinar, E. & Oduncuoglu, M. Hybrid classical and quantum computing for enhanced glioma tumor classification using TCGA data. *Sci. Rep* **15**, 25935 (2025). 40676161 10.1038/s41598-025-97067-3 PMC12271389

[87] Li, Z., Xie, W. & Liu, T. Efficient feature selection and classification for microarray data. *PLoS ONE* **13**, e0202167 (2018). 30125332 10.1371/journal.pone.0202167 PMC6101392

[88] Chen, Z. et al. A machine learning model to predict the triple negative breast cancer immune subtype. *Front. Immunol.* **12**, 749459 (2021). 34603338 10.3389/fimmu.2021.749459 PMC8484710

[89] Ben Azzouz, F. et al. Development of an absolute assignment predictor for triple-negative breast cancer subtyping using machine learning approaches. *Comput. Biol. Med.* **129**, 104171 (2021). 33316552 10.1016/j.compbiomed.2020.104171

[90] Chen, D. L., Cai, J. H. & Wang, C. C. N. Identification of key prognostic genes of triple negative breast cancer by lasso-based machine learning and bioinformatics analysis. *Genes* **13**, 902 (2022). 35627287 10.3390/genes13050902 PMC9140789

